# The future of scholarly communications

**DOI:** 10.3399/bjgp20X712709

**Published:** 2020-09-24

**Authors:** Robert Kiley

**Affiliations:** Head of Open Research, Wellcome, London.

## INTRODUCTION

As the rush intensifies to find ways to treat and manage COVID-19, one thing is clear: researchers, along with their counterparts in industry and the health services, need unrestricted access to the research literature.

However, after more than 15 years of Open Access (OA) mandates, declarations, and discussions, some 75% of the world’s research literature is, on publication, only available to paying subscribers.^1^

Not only is this lack of access morally unacceptable — as much of this research is funded by the public purse — but it also has serious and damaging consequences. A letter in *The New York Times*,^2^ and signed by the Chief Medical Officer in Liberia, stated that the Ebola epidemic could have been prevented had earlier research been made OA, while a study in *Nature Biotechnology* reported that a pharmaceutical company suffered a 6-month setback to a drug development programme because a paper was missed in an inaccessible journal.^3^

## SCHOLARLY COMMUNICATIONS IN THE COVID-19 ERA

Aware of such concerns, and following a global call from science advisors, more than 50 publishers agreed to make all their COVID-19-related content freely available and accessible through PubMed Central (PMC) and Europe PMC. To date, more than 60 000 research articles have been made available through this initiative,^4^^,^^5^ which complements the OA research already published.

Crucially, this content is licensed in ways that support text and data mining and machine learning technologies, allowing researchers and machines to search for and discover new and unexpected connections. One group of scientists have developed a digital coronavirus ‘knowledgebase’ (https://corona.cansar.icr.ac.uk), which uses AI technology to organise large amounts of COVID-19 data as it becomes available.

Perhaps even more significant than making the COVID-19-related peer reviewed content OA, is the way researchers in the life and medical sciences have embraced preprint servers to share their findings as quickly as possible.

Articles submitted to preprint servers — like *medRxiv* (https://www.medrxiv.org) — are screened on submission for plagiarism, non-scientific content, and material that could potentially endanger the health of individual patients or the public, but are NOT peer reviewed ahead of publication. As such, the typical time from submission to publication is around 4–5 days; in the traditional publication model, the typical time from submission to publication has been shown to be around 125 days.^6^

The first article^7^ related to COVID-19 was published on the *bioRxiv* preprint server on 19 January, just 20 days after the Chinese government informed the World Health Organization of *‘cases of pneumonia of unknown etiology ... detected in Wuhan’*.^8^ As of September 2020, the Europe PMC repository has indexed over 13 000 preprints related to COVID-19.^9^

As preprints have not gone through the peer-review process before publication, there will always be some cases where the information is not scientifically robust. But even when this occurs, researchers are using their critical skills to identify these. By way of example, a preprint that suggested there were *‘uncanny’* similarities between COVID-19 and HIV^10^ was withdrawn within 48 hours after researchers used social media to challenge the methodology used.

## MOVING TO AN OPEN PUBLISHING MODEL POST-COVID-19

The COVID-19 pandemic has demonstrated the need to make research OA. However, we need to build on this momentum and ensure that all research, not just research related to the current pandemic, is made OA. There are huge societal challenges ahead of us, for example, climate change, food security, metal heath, and future pandemics, and ensuring that everyone can access research will provide us with the best opportunity of addressing these.

In moving to an OA world we need to be mindful that we do not simply replace one problem, a lack of access to research, with another one, an inability to publish. This concern exists as the prevailing business model for OA publishing is one based on article processing charges, and if you (or your funder) cannot cover these costs, your ability to publish may be restricted.

However, as OA matures, we are seeing the emergence of new business models, such as Read and Publish, Subscribe to Open, and Collective Action, which seek to cover publishing costs from readers as well as those who publish, much like a subscription model, but one where research findings are not hidden behind a paywall. There are also collaborative, noncommercial models (known as ‘Diamond’ OA) where no fees are levied for either readers or authors, with the costs being met by a funder or an institution.

But we need to do more than simply make research OA. The uptake in the use of preprinting needs to be encouraged further, to make sure that research findings can be shared as quickly as possible. But, in parallel with this, we need to ensure that peer review continues to play a key role in assessing research findings — highlighting errors and omissions, and challenging assertions that are not supported by the data.

One approach that seeks to address all of these issues is the open publishing model pioneered by F1000, and now emulated by funder-managed platforms such as Wellcome Open Research (https://www.wellcomeopenresearch.org) and Gates Open Research (https://www.gatesopenresearch.org).

Articles submitted to these platforms are published as preprints, but unlike traditional preprint servers, the peer-review process is undertaken directly on the publishing platform, thus negating the need for the author to seek publication in a journal.

All of the peer-review reports are made openly available, along with the identities of the reviewers, thus adding further transparency to the process. Authors can then respond to the peer-review comments and, as appropriate, update the article and publish a new version of the research. This is subject to another round of review, and so on.

Moreover, as the peer review is performed after publication, the reviewer can focus exclusively on helping the author(s) to improve their article, and not have to consider whether the work should be published or not. The status of any article — for example, ‘awaiting peer review’, ‘version 2; peer review: 1 approved, 1 approved with reservations’ — is made clear to the reader in the article title.

The platform publishing model also encourages all types of research outputs to be shared, including case reports, protocols, and null and negative results as well as research articles. This feature, coupled with a mandatory requirement that authors disclose how others can access the underlying data on which the conclusions were drawn, helps to ensure that others can seek to replicate the research.

At a time when there are significant concerns about the scientific process — for example, recent polls have found as few as 50% of people in the US are committed to receiving a vaccine for COVID-19^11^ — it is critical that research and the underlying data are published in an open, transparent manner.

In a world where all research findings are published under an open research publishing model the role of publishers (especially scholarly societies) will change.

One likely outcome is that they switch from publishing primary research to one that focuses on curating the literature, highlighting which articles published on these platforms are important to their communities and why. In fields like mathematics this is already happening with the launch of ‘overlay’ journals like *Discrete Analysis* (https://www.discreteanalysisjournal.com), which seeks to identify what it considers to be the most important preprints relevant to discrete structures. In the medical and life sciences, *eLife* have launched a ‘Preprint Review’^12^ service, in which submissions bypass the usual editorial assessment and proceed directly to peer review. The peer review reports are published on *bioRxiv*, and if the editors at *eLife* decide the article meets their standards, then it will ‘curate’ that article by formally publishing it.

## CONCLUSION

COVID-19 has changed all our lives and will undoubtedly have profound, long-term effects on our society. It has also shone a spotlight on the shortcomings of the current scholarly publishing system, but also provided an indication of what the future could look like.

Seizing this opportunity and ensuring that all research is published in a fast, open, transparent way, and in ways in which support reproducibility and engender trust, must become one of the positive and lasting outcomes from this global health emergency.
